# Timeout for Contrast: Using Physician Behavior Modification to Reduce Contrast in the Catheterization Laboratory

**DOI:** 10.1155/2019/9238124

**Published:** 2019-01-15

**Authors:** Robby Singh, Marcel Zughaib

**Affiliations:** Providence Hospital/Michigan State University, Southfield, MI, USA

## Abstract

**Background:**

As the number of procedures using contrast media continues to rise, the ensuing complications place an ever increasing burden on the healthcare system. Contrast-induced nephropathy (CIN) is a common postprocedural complication after cardiac catheterization.

**Objectives:**

The purpose of our study was to evaluate the impact of physician behavioral modification on reducing the amount of contrast used during the procedure.

**Methods:**

All patients who underwent procedures in the cardiac catheterization laboratory from January 2013 to August 2016 were identified in addition to the total contrast used during the procedure, the type of procedure performed, and the operator performing the procedure. A new addition was made to the preprocedure checklist in September-October 2013 in the form of maximum allowed contrast for the patient.

**Results:**

A total of 12,118 cases were identified. Across all procedures, the mean contrast used during the 8 months prior to the intervention was 118 ml per procedure. Mean contrast used per procedure for the first year after the revised timeout was 105 ml, for the second year was 106 ml, and for the third year was 99 ml.

**Conclusion:**

A significant reduction in radiocontrast use across all operators and procedures after the introduction of a revised timeout procedure that was seen, which is a change that was sustained over a period of three years. With this straightforward intervention involving physician behavioral modification, patients were exposed to less of the nephrotoxic contrast and were consequently at a lower risk of developing dose-depended CIN and other associated complications.

## 1. Introduction

As the number of procedures using contrast media continues to rise, the ensuing complications place an ever increasing burden on the healthcare system. Contrast-induced nephropathy (CIN) is a common postprocedural complication in the cardiac catheterization laboratory. CIN is a common cause of hospital-acquired acute kidney injury that is associated with a high risk of in-hospital and 1-year mortality [1]. The increased morbidity and mortality from acute renal failure [2] has a significant impact not only on patients but also on healthcare economics.

CIN has been traditionally defined as the impairment of renal function with a 25% increase of serum creatinine from baseline or an absolute increase of 0.5 mg/dL from baseline within 2-3 days of contrast administration in the absence of other causes [3]. The KDIGO-AKI criteria are alternate criteria that have multiple stages of AKI with stage I 1.5–1.9 times the baseline creatinine, stage II 2–2.9 times the baseline creatinine, and stage III 3 times the baseline or increase in serum creatinine to ≥4 mg/dl [4]. The risk factors for developing CIN include diabetes mellitus, congestive heart failure, and preexisting renal impairment and the use of angiotensin-converting enzyme inhibitors [3]. The total volume of contrast administered is directly associated with the incidence of CIN [5].

Numerous interventions have been attempted to reduce the occurrence of contrast-induced nephropathy. Hydration with normal saline is a preventive strategy used to prevent CIN, and the Prevention of Contrast Renal Injury with Different Hydration Strategies (POSEIDON) trial found that left ventricular end diastolic pressure- (LVEDP-) guided hydration significantly reduced the risk of contrast-induced nephropathy compared to standard hydration therapy [6, 7]. The use of N-acetylcysteine had been shown to be inversely associated with the risk of CIN in patients undergoing coronary angiography and computed tomography in a statistically significant fashion, but the protective effect was not seen in peripheral angiography cases [8] though recently the PRESERVE trial did not reveal any benefits of using acetylcysteine to help reduce 90-day morbidity and mortality in patients undergoing angiography [9]. In patients with chronic kidney disease, it was shown that combined sodium bicarbonate and ascorbic acid administration could prevent CIN after catheterization [10]. However, a meta-analysis comparing sodium bicarbonate to 0.9% saline found that sodium bicarbonate did not reduce the risk of developing CIN [11]. And, while hydration with normal saline has been used as a prevention strategy, few studies have evaluated behavioral modifications of operators in the catheterization laboratory prior to procedures.

Khawaja et al. [12] reported a physician behavioral modification study that included systematic monitoring of physicians and communications with physicians regarding total contrast used during catheterization cases. The results showed a significant reduction in contrast usage after the behavioral modification program was implemented. However, the study only looked at cases in which a high volume of contrast had been administered and then informed the physician during the case that maximum calculated contrast dose was reached and by a critical letter after the procedure, which served as a negative reinforcement.

The purpose of our study was to evaluate whether informing the physicians of the maximum allowed dose of contrast, calculated by multiplying the creatinine clearance obtained from the Cockroft–Gault equation by 3, prior to the procedure during the preprocedure timeout had an impact on maximum contrast used during catheterization laboratory procedures.

## 2. Methods

Using the hospital database, data on all procedures in the cardiac catheterization laboratory were obtained from January 2013 to August 2016. During the months of September-October 2013, a new addition was made to the preprocedure checklist in the form of maximum allowed contrast for the patient, as calculated by obtaining the creatinine clearance from the Cockroft–Gault equation, “Creatinine clearance (ml/min) = ((140-age in years) × (body weight in kg))/(72 × serum creatinine in mg/dl).” This equation accounts for the patient's age, weight, and preprocedure creatinine. The creatinine clearance obtained was then multiplied by 3 to determine each patient's maximum allowed contrast dose prior to the procedure, as noted by Gurm et al. [13]. Next, the preprocedure checklist was verified by the catheterization laboratory staff and the operating physician prior to starting each case. Data were collected and included the total contrast used during a procedure, the type of procedure performed, and the operator performing the procedure. For a patient undergoing a left cardiac catheterization with subsequent percutaneous coronary intervention (PCI), the procedure was labeled as left heart catheterization with PCI as opposed to a scheduled PCI alone. The months of September and October 2013 were omitted from the analysis as the revised preoperative checklist procedure was implemented during this transition period. Cases in which no contrast was used or the procedure was not performed were also omitted from the analysis.

However, it should be noted that the maximum allowed dose of contrast was not an absolute threshold but rather a reminder to the physician that the risks and benefits of using more contrast should be considered. The contrast was administered by manual injection for coronary opacification and for left ventriculograms when the systolic function was normal and end diastolic pressure allowed, otherwise the left ventriculogram was done with a device with the total amount, time, and rate of contrast determined by the physician. During the procedure, the physician would indicate to the catheterization laboratory staff if contrast was spilled or wasted and the final recorded total amount of contrast used during the case did account for the wasted contrast.

Overall mean contrast use was determined for the 8 months prior to the intervention, January–August 2013 and then subsequent 1-year intervals after the revised preoperative checklist, November 2013–October 2014, November 2014–October 2015, and November 2015–August 2016. The mean contrast used per procedure for the top 3 most frequently performed cases and top 3 prolific operators at the institution was also analyzed.

## 3. Results

**Figure 1:**
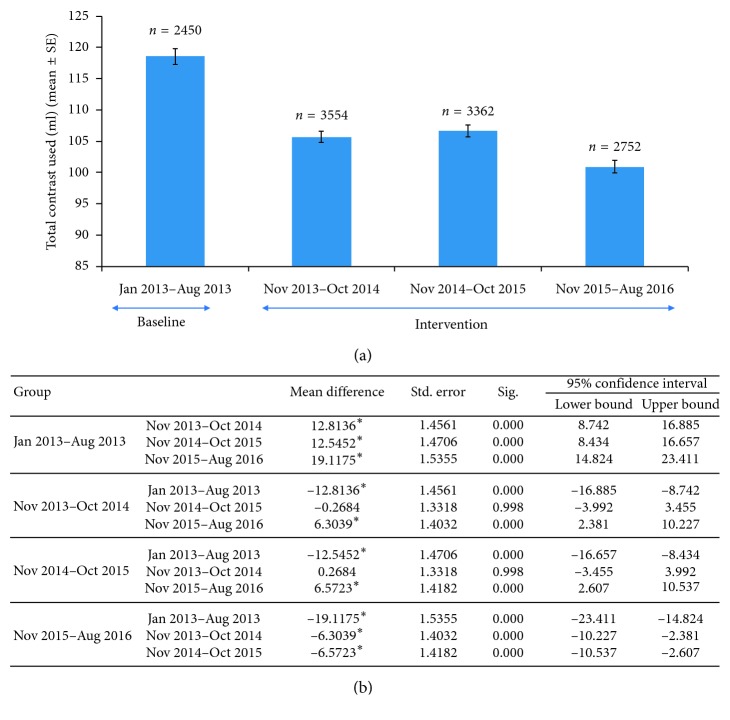
(a) Total contrast use over 4 years across all procedures and operators with sustained significant reduction noted after the revised timeout procedure. (b) Statistically significant comparison of overall contrast use across all procedures and operators.

A total of 12,118 patients were included in the study. The preintervention period was 8 months and included 2,450 patients, while the next two 1 year periods included 3,554 and 3,362 patients, respectively. The final period of 10 months included 2,752 patients (Figure 1). The mean contrast used per procedure during the 8 months prior to the change in preoperative checklist was 118 ml across all procedures performed in the catheterization laboratory. The mean contrast used per procedure for the first year, after the revised timeout was 105 ml per procedure, for the second year, the mean contrast used per procedure was 106 ml, and for the third year, the mean contrast used per procedure decreased further to 99 ml per case (Figure 1). The decrease in contrast in the first 2 years was statistically significant compared to the preintervention period as the further decline noted in contrast use during the third year compared to the previous year.

**Figure 2:**
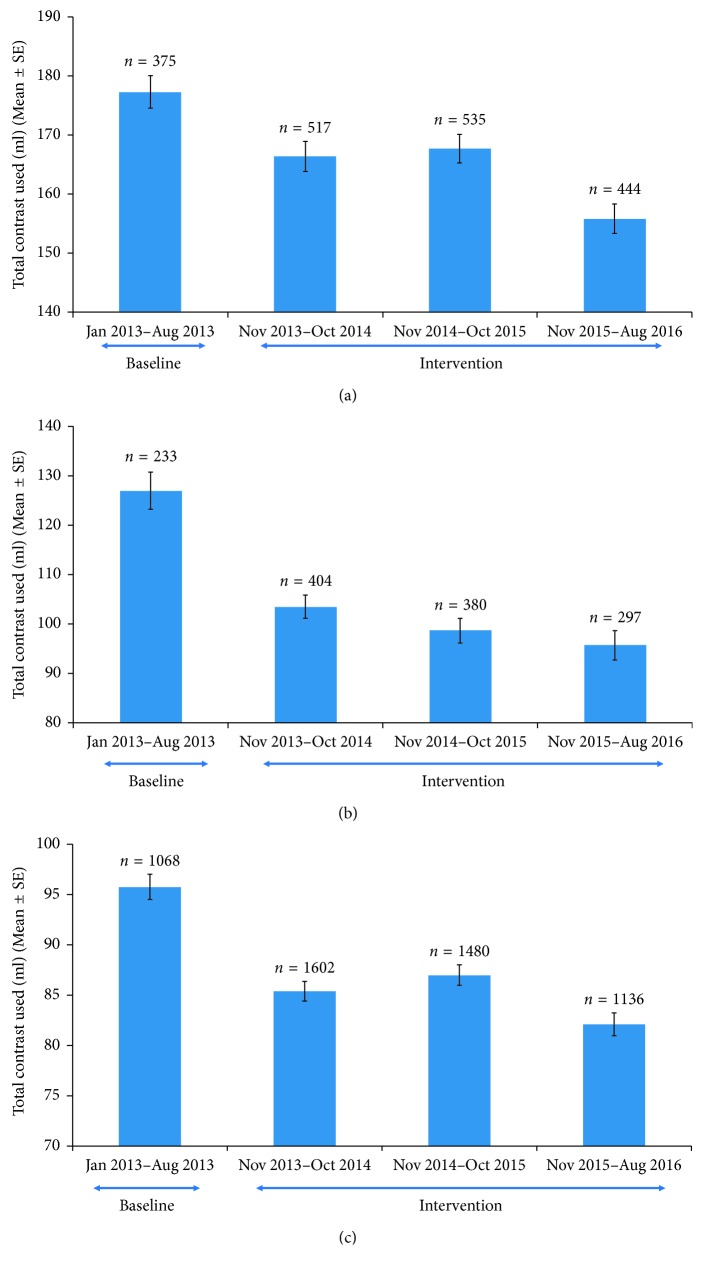
(a) Average contrast dose per year for left heart catheterization and PCI cases. (b) Average contrast dose per year for peripheral intervention cases. (c) Average contrast dose per year for left heart catheterization cases.

**Figure 3:**
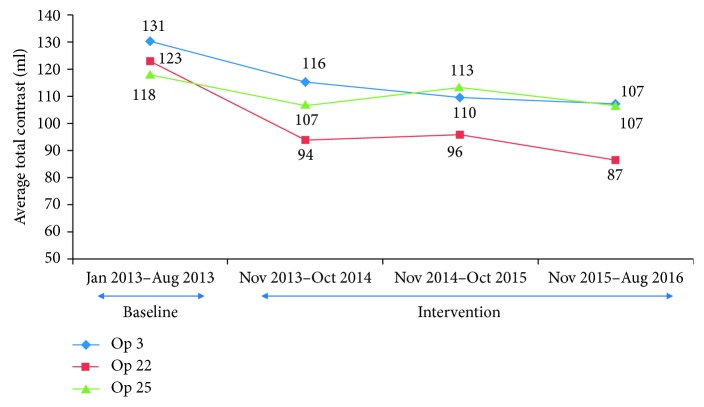
Total contrast use per case over 4 years for the top 3 operators with significant reduction noted after the revised timeout procedure was implemented.

Next, we analyzed the top 3 procedures performed at the institution, which included left heart catheterization, percutaneous coronary intervention, and peripheral intervention. The contrast use decreased significantly in all three procedures compared to the time period prior to the revised timeout procedure, and a similar trend was observed in the third year postintervention having a further decrease in contrast used (Figure 2). Similarly, the top 3 most prolific operators at the institution were identified, and the contrast use per procedure was found to have decreased significantly during the time period as the study as well (Figure 3). Finally, a two-way analysis of variance (ANOVA) was performed with contrast used per case versus provider and time period, and there was a significant decrease in contrast related to the operator and date range. In addition, combined operator and date range showed a significant decrease in contrast (Table 1).

## 4. Discussion

Our study evaluated the impact of a simple physician behavioral modification tool on contrast used in procedures in the catheterization laboratory. Physician behavioral modification remains an underutilized method to help improve patient safety in surgical procedures. While it may initially have appeared to be a minute intervention, simply making the physicians aware of the maximum contrast dose resulted in a significant decrease in contrast used in cardiac procedures. As the institution uses manual injections, physicians have been more cognizant of the amount of contrast being injected during coronary angiography. Not surprisingly, in addition to overall reduction in contrast use per procedure, a subanalysis also revealed a significant reduction in radiocontrast use across the top 3 operators and top 3 procedures performed after the introduction of a preoperative checklist system that included informing the physicians of the maximum allowed dose of contrast for each patient. The reduction in mean contrast per case has been sustained nearly 3 years after the implementation of the revised system.

In addition, it was noted during the analysis that the mean contrast use per case decreased significantly from year 2 to year 3 (Figure 1). Upon further investigation, it was found that a new policy had been implemented during this time that required the attending physician, in addition to the fellow physician, to be present during the timeout procedure. Consequently, an additional overall 16% reduction in mean contrast use per case was seen after the new policy had been implemented.

## 5. Limitations

While our study showed the effectiveness of physician behavioral modification, it was not without limitations. First, our preintervention period was 8 months compared to 12-month follow-up periods. This was due to a technical limitation as the electronic medical record (EMR) prior to our study did not allow for documentation for the contrast used in each procedure. It is certainly possible that some procedures may have been performed more frequently during this relatively smaller time period compared to other time frames. We addressed this limitation by evaluating the average contrast used in the top three most commonly performed procedures across all time periods, prior to intervention and postintervention.

Second, our EMR did not make it technically feasible to determine whether each individual procedure stayed below or went over the maximum calculated dose of contrast allowed. To address this, we observed the mean contrast used per procedure by the top 3 most prolific operators in our institution and noted a similar reduction in contrast use across the operators from the period prior to the intervention and the most recent time period.

Finally, we were not able to assess the incidence of CIN in patients in our study. There are various reasons for this. Most importantly, this is a retrospective study, and checking creatinine postprocedure is not currently a standard of care and, in many instances, patients are discharged home the same day after the majority of cardiac catheterization procedures. Typically, patients will be followed up after 1-2 weeks and a follow-up creatinine level is not routinely done. Also, if patients do develop CIN, they do not necessarily return to the original institution, making the follow-up difficult. As stated earlier, though it may be speculative to some extent, it has been shown in numerous studies that as more contrast is used, patients are at a higher risk for developing contrast-induced nephropathy in a dose-dependent manner [14]. Consequently, the aim of this study was not to evaluate the incidence of CIN, but rather show how a simple intervention can result in significant physician behavioral modification that may reduce the incidence of CIN.

## 6. Conclusions

As has been noted, with these simple and straightforward interventions, patients in the catheterization laboratory were exposed to a significantly lesser dose of nephrotoxic contrast, and the change has been sustained for nearly 3 years. Due to the reduction in contrast use, patients are presumably at a lower risk for developing dose-dependent CIN, based on prior studies. Our study showed how modulation of human factors through checklists and timely reminders can influence physician behavior and result in drastic changes in patient care and safety. Hence, it would be reasonable to attempt similar physician behavioral modifications in other disciplines of medicine and surgery to help reduce morbidity and mortality associated with medical and surgical therapies.

## Figures and Tables

**Table 1:** Operator is related to significant decrease in contrast, date range is related to significant decrease in contrast, and combined operator and date range show significant decrease in contrast.

Two-way ANOVA contrast vs. provider and time period		
	*p* value	Statistically significant?
Cath operator name	<2.2*e* − 16	Yes
Date range	5.009*e* − 14	Yes
Cath operator date range	0.00149	Yes

## Data Availability

The data used to support the findings of this study are available from the corresponding author upon request.

## Abbreviations

CIN: Contrast-induced nephropathy
POSEIDON: Prevention of Contrast Renal Injury with Different Hydration Strategies
LVEDP: Left ventricular end diastolic pressure
ANOVA: Analysis of variance.
